# Retracted: Genetic Analysis and Molecular Identification of Virulence in *Xanthomonas oryzae* pv. *oryzae* Isolates

**DOI:** 10.1155/2014/780801

**Published:** 2014-05-04

**Authors:** ISRN Molecular Biology

This article has been retracted as it is essentially identical in content with the published article titled “Two genotypes of* Xanthomonas oryzae* pv.* oryzae* virulence identified in West Africa,” authored by Onasanya Amos, M. M. Ekperigin, A. Afolabi, R. O. Onasanya, Abiodun A. Ojo and I. Ingelbrecht, published in International Journal of Genetics and Molecular Biology, August 2013 (DOI: 10.5897/IJGMB2013.0070) (see [1]).
